# An exploratory content and sentiment analysis of the guardian metaverse articles using leximancer and natural language processing

**DOI:** 10.1186/s40537-023-00773-w

**Published:** 2023-05-28

**Authors:** Sezai Tunca, Bulent Sezen, Violetta Wilk

**Affiliations:** 1 Faculty of Business Administration, Gebze Technical University, Kocaeli, Turkey; 2 School of Business and Law, Edith Cowan University, Joondalup, Perth, WA Australia

**Keywords:** Metaverse, Content analysis, Thematic analysis, Natural language processing (NLP), Sentiment analysis, Computational qualitative analysis, Leximancer

## Abstract

The metaverse has become one of the most popular concepts of recent times. Companies and entrepreneurs are fiercely competing to invest and take part in this virtual world. Millions of people globally are anticipated to spend much of their time in the metaverse, regardless of their age, gender, ethnicity, or culture. There are few comprehensive studies on the positive/negative sentiment and effect of the newly identified, but not well defined, metaverse concept that is already fast evolving the digital landscape. Thereby, this study aimed to better understand the metaverse concept, by, firstly, identifying the positive and negative sentiment characteristics and, secondly, by revealing the associations between the metaverse concept and other related concepts. To do so, this study used Natural Language Processing (NLP) methods, specifically Artificial Intelligence (AI) with computational qualitative analysis. The data comprised metaverse articles from 2021 to 2022 published on The Guardian website, a key global mainstream media outlet. To perform thematic content analysis of the qualitative data, this research used the Leximancer software, and the The Natural Language Toolkit (NLTK) from NLP libraries were used to identify sentiment. Further, an AI-based Monkeylearn API was used to make sectoral classifications of the main topics that emerged in the Leximancer analysis. The key themes which emerged in the Leximancer analysis, included "metaverse", "Facebook", "games" and "platforms". The sentiment analysis revealed that of all articles published in the period of 2021–2022 about the metaverse, 61% (n = 622) were positive, 30% (n = 311) were negative, and 9% (n = 90) were neutral. Positive discourses about the metaverse were found to concern key innovations that the virtual experiences brought to users and companies with the support of the technological infrastructure of blockchain, algorithms, NFTs, led by the gaming world. Negative discourse was found to evidence various problems (misinformation, harmful content, algorithms, data, and equipment) that occur during the use of Facebook and other social media platforms, and that individuals encountered harm in the metaverse or that the metaverse produces new problems. Monkeylearn findings revealed “marketing/advertising/PR” role, “Recreational” business, “Science & Technology” events as the key content topics. This study’s contribution is twofold: first, it showcases a novel way to triangulate qualitative data analysis of large unstructured textual data as a method in exploring the metaverse concept; and second, the study reveals the characteristics of the metaverse as a concept, as well as its association with other related concepts. Given that the topic of the metaverse is new, this is the first study, to our knowledge, to do both.

## Introduction

Facebook founder and CEO, Mark Zuckerberg, introduced "Metaverse" at the "Facebook Connect 2021" meeting held on October 28, 2021, and, since then, it has become one of the most mentioned concepts in the world [1]. The metaverse is the result of technological process pioneered by the gaming industry and supported by virtual reality (VR) applications, with the possibilities of the information technology (IT) world. It uses extended reality (XR). VR, mixed reality (MR), augmented reality (AR) technologies are under the XR umbrella [2]. It is a virtual universe, a digital world with avatars [3]. Its infrastructure is built on blockchain technology, which has propelled the metaverse to offer anew norm of social networking in three-dimensional (3D) virtual worlds [4]. It is important to analyze the metaverse platform. Every day, thousands of people join the virtual world [5] and billions of dollars are invested [6]. It is becoming the place where people spend most of their time and to which companies have turned their attention. According to Gartner, Inc., it is estimated that by 2026, 25% of people will spend at least one hour a day in the metaverse for work, shopping, education, social and/or entertainment [7]. The metaverse not only replicates the physical world virtually, but also organizes the physical world in accordance with its own structure with digital layers. The digital, virtual world claims to mirror whatever is in its own physical, real world, and even to create what is not. Moreover, it places digital layers on the physical world and manipulates it according to its own virtual world requirements. While doing this, the metaverse transforms its system by applying the rules it sets or that are set for it. It has a structure that can create its own communities and provide its own financing [8]. To fulfill these goals, it uses resources from the physical world and when using such resources, it causes positive or negative effects on the natural world. Therefore, it has become imperative to analyze the interaction between the two worlds, the virtual and the reality. This study analyzes the themes within the positive and negative interaction between the metaverse and the reality. The exploratory study into this interaction and into the characteristics of the metaverse, has referred to the global news mainstream media outlet, theguardian.com, specifically articles published on the topic of the metaverse and analyses this qualitative data with computational qualitative analysis methods.

Notably, the general purpose of exploratory, qualitative research is to provide an understanding of a particular social phenomenon. The data collected for this purpose is generally obtained by the researcher from first-hand observation, interviews, surveys, records made in natural environments, documents, and artifacts. There are various analysis methods and content analysis is one of the qualitative research techniques [9]. It is a very useful method for analyzing textual data [10]. This technique has been used as a method in various research to analyze news media content [11] and social media, user-generated content [12–16]. Advances in AI are also causing changes in the methods of content analysis. The data can be easily scraped with AI software interfaces, such as Orange 3 [17], and analyzed with NLP techniques (such as NLTK, VADER) [18]. The automation of research methods with NLP has led to a shortening of the analysis processes. It has made content analysis more reliable by minimizing human bias in the analysis of unstructured large textual data [19]. Conventional content analysis has been replaced by computational content analysis, through the inclusion of AI [12]. In this study, computational thematic content analysis was performed using NLP methods and AI-based Leximancer program [20]. The unstructured textual data was first collected via the theguardian.com API with the keyword "metaverse” and stored as pdf files for content analysis focuses on thematic and sentiment analysis. Positive and negative discourses about the metaverse included published expert opinions, official statements, company executive statements, employee disclosures and user comments, from the emergence of the concept of metaverse to the time of the study (25 Apr. 2022).

## Background

The concept of the "metaverse" first emerged from the 1992 sci-fi novel by Neal Stephenson, Snow Crash, as a place where people flee to escape a dangerous corporate-dominated world [21]. In the novel, the "metaverse" is a huge shared virtual space made up of all VR, AR, and the internet [22]. Metaverse, as we know it presently, is an all-encompassing term for a mix of physical and digital worlds, where people lead their professional and social lives with a mix of VR and AR [23]. It is the first virtual world [24] that aims to be all-encompassing [25]for an immersive experience that blends the physical and digital worlds with a mix of technologies. It is an online world that can create its own economy [24]. It is a platform used to facilitate social networking and experiences for people [26]. The most important feature of the metaverse is that everything is possible [27]. Stephenson proposes a flattened and rationalized version of our messy and chaotic world [28]. Just as a physical chair is made of atoms and quarks, a virtual object is created using digital processes [29]. Therefore, the metaverse is not defined as being either “traditional” or “non-traditional” [30]. It is a universe that will explore the interface between human experience and AI technology and explore the future of humanity by leveraging Dante's concepts of purgatory and hell [31]. The metaverse, as conceived by technology traders who produce surprisingly diverse artworks, sell NFTs, and use words like "cryptoverse", can only be described as spiritually lacking [32]. AI offers a digital world where people can virtually lead their social and professional lives, allowing customized content to be created to entertain or inform. It is a platform where our real selves will be powered by avatars and AI. It is experienced not only by humans, but also by digital humans [33].

On the other hand, although the metaverse and some concepts related to the metaverse seem to be new, the concept of VR, which is part of the metaverse, has a long history. VR was popularized in the 1980s by an anti-technology computer scientist, Jaron Lanier. His company, VPL Research, short for Virtual Programming Languages, became so successful that toy manufacturer Mattel licensed the "DataGlove" device (a type of wired glove) to create a Nintendo game controller [28]. VR and AR [34, 35], new web 3.0 startup and new entrepreneurs have spawned new worlds [36, 37], despite the startling and misperception of movies like The Matrix, Terminator, Tron. Meta enters this industry with well-established players such as Roblox and Decentraland and crowds them out [38]. The metaverse base is created methodically and collaboratively with a wide variety of participants [39]. For example, Facebook had rebranded in late 2021 to the new name ‘Meta’ to claim first mover advantage in the metaverse space, and it is focused on acquiring the communities and content of Activision, which are two key parameters for metaverse success [40].

The metaverse is using VR, 3D internet, and AR adding layers of digital imagery to the real world to create new worlds [30]. It evolves with VR/AR. It offers a way to experience the partly virtual and partly physical world with digital objects or text placed over or within the visual field. AR may initially be more effective than VR and completely replace screen-based computing [29]. Some envision the metaverse as a hyper-verse world where people virtually lead their social and professional lives through VR headsets such as Facebook's Oculus Rift and AR where a digital overlay is placed on top of real life [41]. In this universe, avatars (or digital representations of people) meet in the virtual world through its users (people) wearing VR headsets in the real world. Thus, managers now hold weekly team meetings in the metaverse through the company's Horizon Workrooms product [42]. Notably, according to Chalmers' predictions, within a century we will have virtual realities indistinguishable from the non-virtual world [29].

However, the metaverse claims to be an alternative to the physical world. It has its own laws. With its decentralized structure, it can create its own assets and riches. By sharing these, people can create income belonging to their own universe. The metaverse democratizes its unique social system by forming with its users' councils and boards. It has a circular economy in its evolution by selling digitally generated assets. While fulfilling these facts, it aims to transform the world as we know it by building a bridge with the physical world [8]. However, there is no comprehensive study examining the positive and negative effects of the metaverse on the physical world while achieving these important goals. The new universe, the metaverse, is just emerging in literature. Studies on the metaverse are limited, scarce and very new. Therefore, this study aims to explore the concept of the metaverse, and to reveal which themes, and therefore, which metaverse-related concepts emerge and what effect they may have on the reality as we know it.

NLP, one of the sub-branches of AI, is used in the analysis of unstructured textual data. Software interfaces, such as Orange 3 [17], Leximancer, and Monkeylearn [43], are made for these processes. With software applications such as these, big data consisting of text [44], are automatically classified by Sentiment, Role, Business, Events, and others, for further analysis. In this study, an unstructured textual dataset was created by collecting metaverse-related publications from theguardian.com website with Orange 3. Leximancer was used for the thematic analysis of key concepts, clusters and relationships, and identification of positive and negative sentiment. Further, Monkeylearn was employed for analysis of “role industry, events, business classifiers” by going through the steps in the Metaverse textual dataset working model.

*Leximancer* is a well-known, easy-to-use text analysis tool [45] often employed for computational content analysis. Leximancer detects key concepts (or words) based on their similarity and association with other words within blocks of text in the complete dataset. It used machine learning to automatically generate and classify its own glossary for each set of data. The classified texts are indexed as main themes, concepts, and compound concepts. A conceptual mapping is generated by determining the relationships between the indexed theme and the key emergent concepts. As in previous studies of similar nature, the conceptual structure of many articles in the present study’s dataset was summarized, indexed, measured and visualized [46, 47]. Leximancer, allows computational content analysis for qualitative data analysis, and determines the frequency of the concepts, the association between the concepts, and the closeness of the co-occurrence of the concepts [48]. It has been used in many academic studies [40–44]. One of the issues in research with NLP is research bias, either caused by human error or through the researcher’s limited epistemological position. Such bias is minimized through the use of software such as Leximancer, which automatically performs the required thematic content analysis of textual data. This increases the validity of the research [49]. In this study, Leximancer 5.0 version was used to analyze theguardian.com “metaverse” articles.

Leximancer contains many features, including the Leximancer Topic Guide (LTG) [20]. LTG represents a new technology to automatically generate a story list and topic index for a large collection of documents. LTG is a tool for reviewing large reports or collections of documents. It is designed to make the review of analyzed large articles more efficient and more effective. It provides the opportunity to select relevant topics in the large text by specifying which topics are required. Thus, the topics are interpreted in accordance with the research purpose or converted into a dataset for another analysis. In this study, LTG enabled the selection of metaverse-related article contents in accordance with the research purpose. Sentiment analysis was undertaken by converting the contents selected from LTG into a dataset. The sentiment analysis was then interpreted as either positive or negative according to the output.

*Sentiment analysis* is a type of classification method used in the perceptual analysis of unstructured textual data [50]. There are different sentiment analysis methods used in NLP. One such method is Valence Aware Dictionary for Sentiment Reasoning (VADER), which is available in the Natural Language Toolkit (NLTK) library. VADER is sensitive to both polarity (positive/negative) and intensity (strength) of perception. VADER works with unlabelled text data [51]. It has been used in many previous research studies [18, 52, 53]. In this study, sentiment classification (positive/negative/neutral) of texts was made by directly applying VADER coded with Python to the textual data set selected through LTG. The compound score of each content was automatically calculated by VADER. The text was interpreted and analyzed based on the calculated score. Then, with Monkeylearn AI attributed compound score, it was classified into separate tables (Tables 2, 3, 4).

*Monkeylearn* is an AI platform for text analytics. It automatically extracts relevant data from unstructured raw text. Easily actionable results are obtained with text classification (Tagging, Categorizing) and extraction models [54]. It has been used in various previous studies [55, 56]. In this study, it was used to classify the metaverse articles. The text was automatically classified according to the content of the texts as “Role Industry Classifier”, “Event Classifier”, and “Business Classifier”. Thus, the role, event, and business class of theguardian.com metaverse contents were determined, followed by the interpretation and representation in tables (Tables 2, 3, 4) according to the scores obtained from the sentiment analysis.

## Methods

### Data collection and framework

**Fig. 1:**
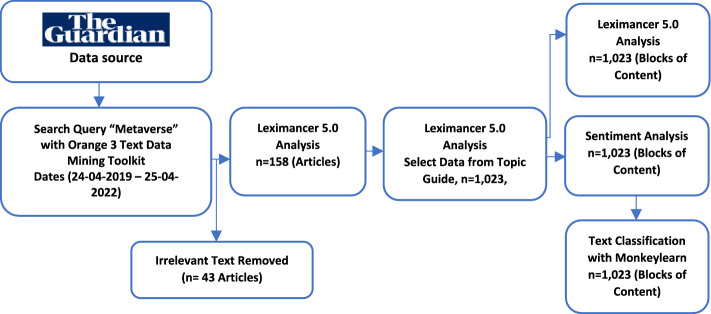
Structure and process of the *theguardian.com* text dataset analysis

**Fig. 2:**
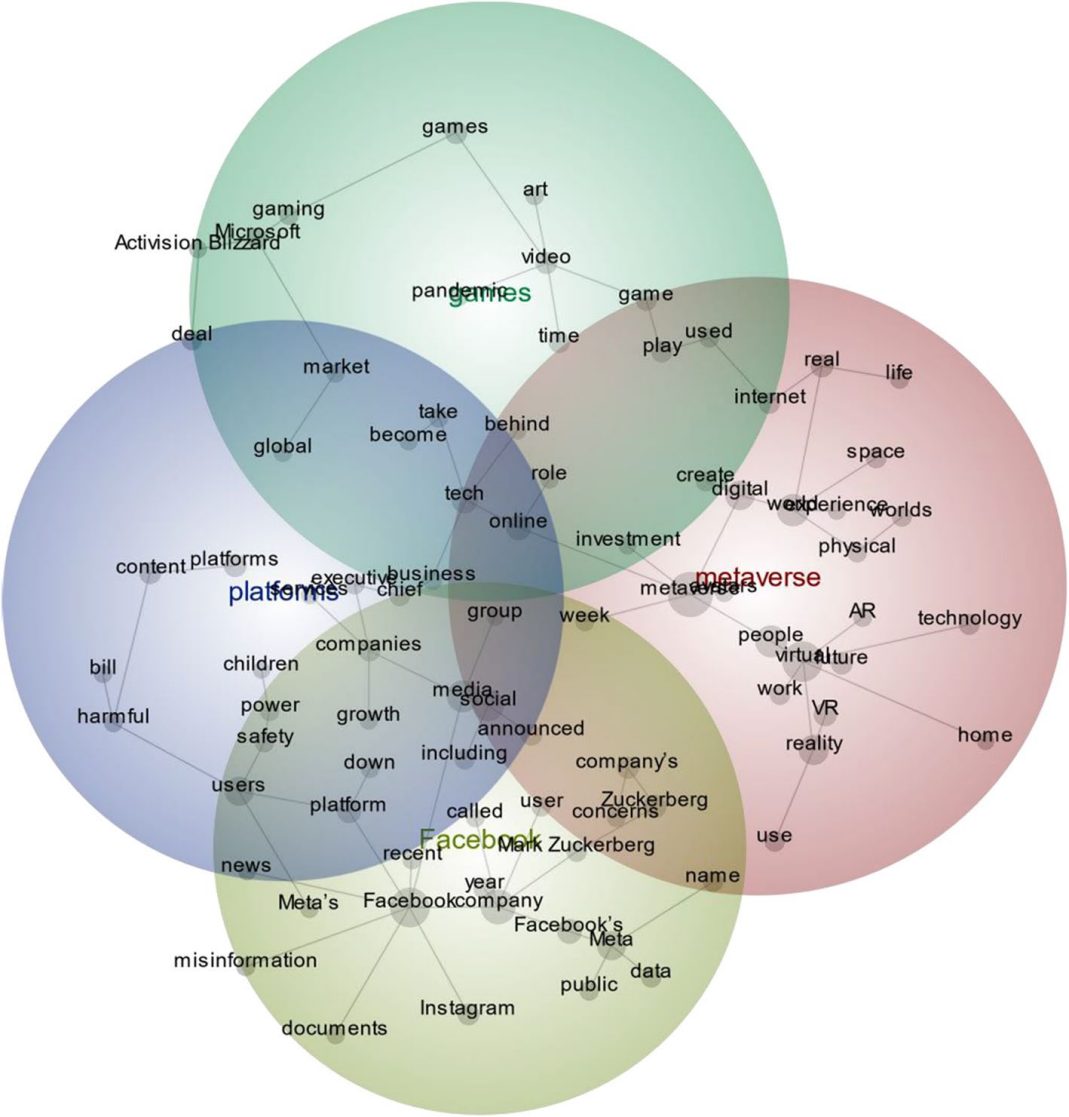
Leximancer concept map (LCM) of theguardian.com Metaverse articles showing the emergent themes, concepts, and their associations

Membership registration with The Guardian (theguardian.com) Open Platform fostered the collection of the required data in the form of articles about the metaverse. The “Metaverse” research keyword was used between 24-Apr-2019 and 25-Apr-2022 to identify the relevant online articles, as shown in Fig. 1. This process entailed the use of Orange 3 Text Data Mining [17] feature using register developer key given by The Guardian API [57]. As a result of the query, 201 articles were identified (n = 201). The textual data obtained from the query was saved as a dataset. When the recorded data were checked, 43 articles were excluded from the analysis because some were deemed irrelevant. Following this data purification process, Leximancer was used to perform thematic content analysis. This entailed selecting only the “Content” sections of the articles (n = 158). Textual contents (or blocks of text) relating to the research topic were selected with the LTG formed by the Leximancer analysis. A new dataset (n = 1023) was created again with the metaverse topics summarized as titles. Purpose-selected sections of the data were recorded for NLP classification analyses. In this way, research reliability was ensured by selecting textual content which related to the metaverse research topic. The new dataset (n = 1023) was used in the Leximancer analysis again and resulted in the data visualization output, a Concept Map, which presented the emergent themes, key concepts, and relationships (associations) between them (Fig. 2). Following this process, the textual dataset was coded with Python, using the VADER method, and the text was classified according to sentiment type (positive/negative/neutral). According to the sentiment analysis results, the contents were evaluated one by one and summarized as positive and negative. Finally, the analysis was completed by using Monkeylearn classifiers (role, business, and events) to classify the data according to the sentiment score.

## Analysis and results

### Leximancer analysis results

The Leximancer Concept Map (LCM), in which Leximancer analysis results are visualized, shows the key concepts which appear most frequently in the text, and which are most closely related to other concepts in the map (Fig. 2). In LCM, concepts are clustered into higher level "themes". In the analyzed text, the concepts are strongly associated with each other, based on according their frequency and coexistence. Themes tend to be located close to each other in the Concept Map and are shown as colored circles on the map [35]. The size, intersection, distance, proximity, and relationship of the colored circles indicate the most prominent overarching concept clusters. The LCM was set to display the first 33% of concepts. Thus, the LCM of the metaverse articles (Fig. 2) indicated the following themes: “metaverse”, “Facebook”, “games”, and “platforms”. According to the results, “metaverse” (red colored theme) was the most prominent and highly connected theme (cluster of concepts) in the articles analysed in this study, while “platforms” (purple colored theme) was the least connected theme within the articles. The central, key concepts within the "metaverse" theme were the concepts of "metaverse", "virtual", "digital", "world", "reality", "people" and "internet". When the prominent conceptual relations in the "metaverse" theme were examined, it was revealed that "metaverse-virtual", "metaverse-digital-world-physical", "digital-world-experience", "virtual-future", "virtual-reality-use", "digital -create” was “metaverse-avatars”.

“Facebook” (yellow colored theme) formed the second important cluster of concepts emergent from the metaverse articles. The core concepts within this theme, were “company”,”media”, ”platform”, ”user”. The important conceptual relationships in this theme were "facebook-platform-down", "facebook-misformation", "platform-users-safety", "facebook-meta-data".

“Games” (green colored theme) was the third most prominent theme within the analysis. The most important concepts here were “video”, “Microsoft”, “games”, “tech”. The prominent conceptual relations were “video-art”, “video-pandemic”, “video-games-gaming-Microsoft”.

The following prominent theme was that of “Platforms” (purple colored theme). The core concepts in “Platforms” were “users”, “content”, “tech” and “companies”. Conceptual relations which were noteworthy were “platforms-content-harmful”, and “users-safety”. The metaverse textual analysis results of these findings, visualized with LCM, were important in terms of demonstrating and identifying key metaverse concepts, clusters, and their associations. In qualitative research, pre-analysis research concepts are expected to be related to each other. It can be said that the LCM results were coherent and represent concepts related to the metaverse in a consistent way. After this verification, sentiment analysis, which is the next step of the study, was commenced.

### Sentiment analysis results

The VADER sentiment analysis (Table 1) of theguardian.com metaverse dataset (n = 1023), which was created by selecting from LTG, identified positive (n = 622) 61%, negative (n = 311) 30%, and neutral (n = 90) 9% sentiment. Consequently, this analysis showed that the sentiment about the metaverse is predominantly positive.

**Table 1:** Metaverse sentiment analysis results

Row labels	Count of content	
Positive	622	61%
Negative	311	30%
Neutral	90	9%
Total	1023	100%

Further to this analysis, the contents were examined and explained under separate headings and subheadings as positive/negative. Subheadings that constituted positive content, included: *Games and players, Blockchain, NFTs, Algorithms, data, Companies and Business, Technology, equipment, children, youth, people, society and culture, users, avatars, experiences, security, and equipment*. Negative content included: *misinformation, harmful content, algorithms and software, data, equipment, children, women, people, artists, and society*.

### Positive contents

Despite claims of monopoly [58], the metaverse continues to lead the industry in all categories in which Facebook competes [59]. Some have questions whether virtual worlds, as they appear now, controlled by corporations, lead to potentially dystopian realities where corporations control everything in our environments [60]. Despite the prediction that some of the policies will not change unless the metaverse managers change [61], some have pointed out that with the Meta brand change, Facebook will improve its targeting and measurement techniques and will generate more revenue from its users [62]. Notably, the use of the metaverse has increased 12 times since 2020 [63]. It continues to offer services and hardware to developers at low or no cost to attract a critical audience [64]. Meta's apps, including Facebook, Instagram, and WhatsApp, are used by 2.8 billion people every day (Insert reference here). It is claimed that people are forcibly pushed onto these platforms [65]. However, the company does make public statements about its products and work [66]. Facebook has prioritized the metaverse platform with the motto "From now on, we will be metaverse first, not Facebook first" [67].

Another player in the Metaverse ecosystem, Microsoft wants to control as much of the gaming market as possible in the future, and it aims to do so by investing in content rather than hardware [68]. Microsoft's metaverse vision is described as "the cherry on top of the market" [25]. By investing 10 billion dollars for the metaverse and Microsoft 70 billion dollars, they have clearly indicated that they want to be the heart of the games metaverse [69]. Microsoft is also creating virtual layers over existing urban infrastructure where people can use mixed reality lenses, such as glasses or contact lenses, to interact with each other [25].

#### Game and players

Gaming is the most dynamic and exciting category in entertainment across all platforms today and will play an important role in the development of the metaverse [40]. Virtual Reality (VR) centres around immersive environments, and the gaming industry specializes in creating just such environments [70]. With the metaverse, games began to go beyond the concept of games. It makes games a part of the environment [71]. The endless possibilities of video games allow for interesting experiences. People maintain their personal and professional lives online via VR headsets or Pokémon Go-style AR [72]. In the metaverse, virtual games become a digital venue [36] that will enable an online community to, for example, gather around major sporting events, from the NBA playoffs to the soccer World Cup. Time spent can include furiously roaming the in-between lands on a ghostly horse, killing trolls, exploring castles, and being branded over and over by giant boss monsters [68]. From in-game parties [73], online golf games [68], to golf ball-eating pomace [74], full of interesting innovations that will surprise players at any moment, there are many metaverse experiences. Wearing a VR headset and tactile clothing, one can drive a flying car to a perfectly simulated mansion in a soothingly sanitized alternate reality [32]. Added to all this, music plays a big role in virtual environments [75]. This makes the metaverse a platform that can make the game safe, inclusive and accessible to everyone [40], while also offering fun, different and incredible experiences.

Even in the pandemic, with stores closing and dressing opportunities evaporating, big brands have rushed into existing online worlds, such as video games, and developed their own VR and AR experiences [76]. The gaming industry has evolved into an international retailer selling luxury fashion houses and virtual clothing tailored to the user's image. They have raised capital through cryptocurrencies to be able to invest in the metaverse development of game companies [24]. With B2B [business-to-business] gaming platforms [77], the virtual world has become a safe haven for people and companies in the metaverse [60] from environmental impacts such as the pandemic. Metaverse users see games and virtual worlds as an escape from the inequalities and injustices of the real world [32]. At the same time, “play-to-earn” crypto [78] creates users’ own economy by the ability to generate their own earnings in games with monetary incentive systems [59], producing crypto money [78]. Players expect metaverse games to have no menus and button layouts that are part of the physical world that compels the player [79] and instantly translate conversations between players in different languages [80]; creating a seamless, satisfying metaverse experience.

#### Blockchain

Blockchain forms the infrastructure of metaverse technology. The metaverse versus the highly centralized physical world plays an important role in the transition to the decentralized system with its positive effect on the crypto market [28]. The crypto market has become the American Gold Rush with the influence of the metaverse, individuals meet the blockchain world [81]. The cryptocurrency market is currently valued at more than $3 trillion, designed to allow it to send online payments anywhere in the world without government oversight [79]. It is that both centralized (tech giants influencing powers and rival nation-states) and decentralized parties (crypto innovators who remain an influential subculture) see the new trend in technological advancement in roughly the same terms [28]. New concepts like cryptocurrency/blockchain capitalism [62] are getting stronger.

#### NFTs (non-fungible tokens)

Non-fungible tokens (NFT) became more popular with the metaverse. NFTs are stored in a decentralized transaction ledger on a blockchain, which is the same technology used to buy and sell cryptocurrencies [82]. NFTs can be used for art, video clips, music, and many other applications. An NFT is a unique digital asset that represents the ownership of real-world items [83]. Collins defines an NFT as "a unique digital certificate registered on a blockchain used to register ownership of an asset such as a work of art or a collection" [63]. In the metaverse, NFTs are used to register ownership of just about anything, including digital art, music, movies, games, and pornography. Once files are uploaded and verified by a third party, they acquire a rare status, much like a hard-to-find stamp or a unique piece of couture [82]. For an NFT, each coin is unique. This makes NFTs popular to register blockchain "ownership" of a particular piece of digital art or memorabilia [84]. NFTs thus give ownership of a unique digital item (whether it's a virtual work of art or a jacket to wear in the metaverse), even if that item can be easily copied. Ownership is recorded in a digital, decentralized ledger—the blockchain [85]. Recorded NFTs are units of data that are unique, inimitable, and non-interchangeable on the blockchain, and can be associated with photos, videos, audio, and other forms of art, allowing these assets to be uniquely and permanently identified [81]. With existing crypto tools, keys are created that would take millions of years for traditional computers to crack [86].

NFTs appear everywhere from art departments to finance pages, galleries, and auction houses to social media platforms [63]. Considering that it is not so easy to manufacture digital products in the Metaverse, the complexity of production [87] and the cost of NFTs [88], designers want to see them make money and create an economy in the digital world [76]. Although there aren't many directors who positively portray an online world [34], in the metaverse, digital artists create a virtual collection that gets the participants to wear it as well [89]. For example, the Digital Fashion Institute created an NFT dress to be sold. Moreover, one of the biggest moments of London fashion week was performed simultaneously on a runway at Tate Britain and in the metaverse [87]. In fact, instead of buying fast fashion online and taking it to the charity shop a few months later, people can easily exchange clothes with friends and neighbors in "community wardrobes" and virtual closets [90]. Moreover, users easily experience different clothing styles regardless of gender [71].

People get closer to popular artists at virtual concerts [75]. They are even able to obtain a virtual signature [91]. Potential fans can find each other easier in the metaverse and, as such, they create virtual communities. The metaverse users can generate different revenue streams [92] with fan-informing books, a magazine, and online content. Many people will lose big if people buy things from the metaverse simply because they think someone else will buy more [93]. Despite the negotiation, some people have already bought virtual properties from the metaverse [24]. Some have already started making a digital artwork collection [63]. The metaverse also undertook the vision [94]of creating the most open, artist-friendly ecosystem in the physical world. A way has been created for artists to take advantage of incredibly creative and powerful tools [95]. People use machine learning to teach painting [96], buy new clothes that didn't need to be physically produced [97], and create different avatar groups to make creative artworks as NFT [93].

#### Algorithms

A platform's algorithm is designed to encourage greater user engagement in any way possible, including by sparking disagreement and rewarding anger; thus it is important to identify what potential harm the algorithms can do [98]. This issue was brought up by Facebook's integrity team, who suggested changes to the algorithm that would suppress rather than accelerate hostility between users. Experts and editors also express their opinions on what algorithms should entail. It is believed that algorithms direct users to harmful misinformation and give users more chances to shape their online experience [79]. There should be more transparency in how algorithms work [62]. Platforms should inform users about what the algorithm is doing to increase reliability [72]. It is understood that algorithms are fostered by tracking, and profiling users provide positive results by providing more interaction for business models [99].

#### Data

The metaverse doesn't just react to old world data, such as someone’s age and gender, for an advertiser targeting specific audiences in the virtual world. A person’s body language, physiological responses, pave the way for knowing with whom and how you interact [21]. With devices that can read your "emotional data" [90], new types of data, such as biometric data, that have not been widely known or used are now being utilized with the aid of metaverse equipment. It is said that data collected by metaverse elements such as VR [100], which are thought to help determine behavioral norms, will be become increasingly important.

In response to backlash against Meta's unauthorized use of data, it found ways to target users with less data [23]. Facebook agreed to delete all the data it had ‘accidentally’ collected [101]. Mark Zuckerberg declared that for each potential future application, the intended use will remain public on how people can have control over these systems and their personal data, and how the technology lives up to its framework of responsible innovation [102]. It is said that how the data will be used [90] has created a new model for independent academic researchers to access data securely [103].

The immersive new online world controlled by a company with a history of spreading misinformation, collecting data, and spreading harmful body images, has a sense of dystopia due to Facebook's past practices. These past practices along with sci-fi movies on the topic, have contributed to people feeling paranoid about our metaverse future [34].

Seeing data as a key factor in developing AI programs, it is said that "Companies have all this data that becomes a wealth of information that can help make the company more efficient" [33]. Facebook's advertising system relies on data from its users. That's why regulators feel that users should be given more control over this data [104]. Contrary to past applications, the metaverse has a new AI model that can learn from trillions of examples, understand hundreds of languages, and more, with its AI supercomputer called the AI Research Super Cluster (RSC). It is RSC's fastest computer of its kind. AI mimics the basic architecture of the brain in computer form and can handle vast amounts of data. It can detect patterns in the data [80]. Thus, the metaverse uses a new business model that can create its own economy, not just in terms of advertising.

#### Companies and business

The metaverse has created an online space built by creators and developers, where people can live their lives, attend performances, and even work virtually [105]. For such reasons, big technology companies are investing in the “metaverse” as the next big stage for their growth [77]. The Metaverse will create a lot of value for many companies but will require significant investment over many years [59]. VR and AR world development will require continued investment in capabilities across the enterprise [41]. It is the most plausible way for some companies to ascend into the metaverse for their future survival strategy [94]. Also, the metaverse is an opportunity for companies to acquire a unique asset that can drive their consumer strategy forward [40]. For example, conglomerates producing luxury products have started to exhibit their products with VR [76].

Companies are exploring new ways to monetize their user base [106]. It is therefore desirable to take an enormous share of the free time and money of people all over the world [107]. For example, some companies make millions, then billions, by selling virtual clothing and items to gamers eager to embellish their virtual selves. It has its share of people [32] who meet in digital worlds and marry in the real world, have built some of their most important relationships, and have meaningful life experiences [24]. It creates opportunities for companies in important events in the physical world. Big tech firms are the biggest economic winners from the pandemic as global lockdowns push more businesses and consumers to use their services [108].

Metaverse will allow users to customize their avatars and digital spaces, decorate a digital office with images, videos and even books. It allows users to invite their friends virtually, even though they are in different parts of the world, to make remote business presentations of their colleagues [109]. Meetings take place in the office metaverse filled with virtual desks and whiteboards [110]. Users can use gestures to interact with colleagues, move around in their virtual space as if they were in an office, and sit face-to-face around the desk as if they were in a 3D meeting room [111]. In the future, the use of avatars in meetings will be used in all applications [42]. Many companies are adjusting their onsite services and facilities to better reflect hybrid workforce needs. It is predicted that people and teams will be more and more dispersed in the future and therefore attempts are made to create a metaverse experience [112]. Business models are adjusted to provide a multitude of opportunities by abandoning the basic business models over time and moving towards the metaverse [113]. The change in these business models has accelerated during the pandemic. While also fueling concerns about the potential social consequences, change is expanding rapidly across industries, from healthcare to business to entertainment. The effort to create contactless services is designed to increase productivity and reduce bureaucracy [91]. For example, using the digital medium, it is possible to take ones collection anywhere, despite being in quarantine and unable to travel [76]. With an increased focus on climate change and sustainability, polluting business travel has now decreased [58] as activities with a low carbon footprint [114] yield positive results.

All these purchases and sales increase the need for new technologies and platforms in the digital world [81]. Metaverse, a digital transaction world that will create trillions in wealth, presents new monetization schemes for investments [115]. If you create a product, you will be able to create your own business [81]; and with more diverse monetization methods than before [93] new ways to make money [62] with new brands created [37], you will be able to expand your existing business by creating your own business. The metaverse has necessitated thousands of job postings to create new high-skilled jobs. Besides building the European metaverse [116], it has created 10,000 new jobs in the European Union [41]and introduced new occupations and roles all over the world. For example, employees “metamates” [101] for their own employees, a virtual teacher [89], new jobs such as digital artist, new roles such as chief futurist [58]. Moreover, in the metaverse, virtual goods and commerce [109] has become the intersection of consumer culture and real life [117].

#### Technology

The metaverse, is where digital worlds are blurred [118], it is the new concept of technology [42], which is a mixture of physical and digital worlds in which people can interact virtually. The successor to the mobile internet, and a kind of “embodied internet” [28]. With the 3D internet, it can take digital storytelling and place it in real physical spaces [30]. It is the next evolution of the Web 3.0 field or internet [37]. With new immersive cyber technologies [29] such as the networked ecosystem [119], a person’s digital and physical self can be customized. It is the only product in the world that allows someone to decorate projections such as Star Wars-esque 3D holograms [90, 120].

#### Equipment

Smart glasses with AR layers [35], smart lenses, virtual headsets [32], two cameras, a microphone, speaker, and voice assistant that can display via phones [105] are all equipment that fosters the metaverse use. Metaverse equipment improves people's quality of life with a patented technology [32] that can monitor what a person is looking at and how a person’s body is moving in VR. They believe that, without the intervention of the smartphone [26], in the future the internet will take on an even greater role in people's daily lives and instead of interacting with the Internet via mobile phones, people will be immersed in VR headsets [21]. All these require AR glasses to be light, trendy and contain various technical features [35].

#### Children

Hesitations about how harmful virtual environments and platforms can be to children's mental health are unreliable for children unless they provide the safety services they promise [39]. Companies are invested in ensuring that the concept is committed to responsibly building the metaverse where under-13 s are denied access, against their claims [121]. One implementation is the new parental controls for VR headsets, including the ability to block access to certain apps for parents [122]. While sympathetic victims (girls with eating disorders, religious minorities, public figures) take a stand against a weakened Facebook [123], children, shaped by the digital world rather than parents, design everything in their virtual worlds themselves [117]. The future of children growing up with the metaverse is shaped by its creations [34], while gaining new abilities [90].

For teens, hanging out in the vibrant, fun online world can be an experience that fulfills the idea of a massively engaged online metaverse. For a generation of teenagers who are eager to go wherever their parents cannot or will not follow them, the metaverse is a great opportunity [73]. A new generation of musicians and music lovers who grew up in virtual worlds [124] can socialize with their friends, do their work or take part in a video game in the metaverse [25]. People can earn money while doing all this. The result is a younger, aspirational customer base in the metaverse [125].

#### People

The metaverse is a world in which humans can be fully immersed [92]. It is the environment where people can enjoy superhuman powers, have other bodies, experience new sensations, and explore environments with different laws of physics [60]. It is fun, exciting, helpful to people for productivity at work [105]. Like reminiscence therapy [126], it rekindles forgotten memories of youth, enabling the older generation to act like the younger ones. People who become lonely in the real world can join new friends and new groups in the virtual world according to their interests [71]. It refers to individuals who do not have any physical presence, but who are "only engaged in creating their own profiles in the metaverse" or in the virtual public space [82]. People who have more money and time than they used to have, looking for a way out of boredom, are shifting into the metaverse [88]. They are becoming virtual places where people spend most of their time [105], spending their time and money [127]. In the long run, it is predicted that people will spend most of their lives in VR [60]. Because no matter what you do, the metaverse offers a fascinating opportunity for people to be involved in a family-friendly community [36]. This virtual world and its technology can reach a point where the virtual and physical sensory are the same, and people live good lives in VR [60].

#### Society and culture

Social media platforms are becoming key components of the everyday life [119]. Meta and Google are becoming the infrastructure of the digital public sphere [99]. With the introduction of the metaverse, the concepts of “internet community” [34], “a social VR platform” [128] and “online community” [34] became stronger. An exciting virtual environment is formed [32], where the hierarchies and limitations of the real world disappear, where rich-poor, coward-heroes meet in one place and reconcile, away from reality. As virtual worlds become richer and more believable, virtual societies have started to form. Virtual jobs are being undertaken, with the motivations, desires and goals that arise in these types of virtual environments. By creating unique value for virtual communities [94], the metaverse is becoming a fun and harmonious community [129], and it will grow as the number of users increases and the structure follows virtuous policies [27].

Modern digital culture [105] and virtual populations are exploding [34]. It should help to embed western values ​​such as freedom of expression, privacy, transparency and the rights of individuals into the daily functioning of the internet [41]. Organizations such as Human Rights Watch rightly argue that tech companies should conduct comprehensive assessments of how human rights may be harmed before releasing their products to vulnerable parts of the world [127].

It is optimistic about the metaverse's power and reach for virtual experience, despite the risk of the metaverse population being unbalanced in terms of socioeconomic, gender, and ethnicity [105]. With its capacity to create hybrid physical-digital experiences [30], it is on the way to develop this culture. Many people will be able to immerse themselves in culture, learn and understand more using this type of technology [76]. The metaverse will create its own culture with its extensive online metaverse experience [73].

#### Users

A large portion of the metaverse’s revenue is generated by creating profiles of its users that can be matched with advertisers' needs (Insert reference here). When an advertiser is looking for a consumer in a certain location, with a certain demographic and lifestyle profile, metaverse platforms, such as Meta, can direct those ads to the right target audience based on the data it collects about them [23]. However, the metaverse can create its own financial resources. For this reason, routing has become possible with very little user data. With the experience gained from Facebook, the metaverse participants are developing business models that will no longer need user data. Taking care to protect users from harmful content [98], toxic users are removed from platforms [130]. A more reliable environment is provided by closing anonymous accounts. As with other SM platforms, it imposes penal sanctions on users and administrators [131]. Meta, for example, introduces new security measures [132] that encourage users to take a break if they use the app for a long time. The number of Metaverse users is constantly growing with the addition of new users [133], and users inviting friends into their digital worlds [109]. Users can also create their social capital based on interactions and shares within their networks. For this reason, the metaverse user does not need physical world acceptances in order to create their own social capital [82].

#### Avatars

In the metaverse, people represent themselves as avatars (digital representations in VR) [76, 134]. The metaverse allows users' avatars to perform actions such much as one would in the real world, such as reach out, jump, and spin around [135], be part of a group by interacting with everyone seeing each other's avatars and art. People use avatars to move and communicate even if their bodies are far away in the physical space [58]. Currently, the avatar can travel with up to 20 people and play games, hang out and create special digital environments [100]. Avatars have a status in the virtual world and have unrestricted access in online communities within the virtual reality [89].

#### Experiences and feelings

People will get used to living in both a virtual and a real world with the metaverse [24]. Users will feel that they are with each other and will have a “sense of presence” despite being far away [64]. At the same time, they will meet many new experiences and new feelings that they have not experienced before. Some reported examples include: *“It just felt more real *[71]*. It was a composite of digital pictures *[16]*. The laughter was much more sincere than people imagined *[136]*. It makes you feel like you are in space. You are just like a soul; you wear glasses, you are in a beautiful place, but you are not *[89]*. No matter how far away we are, we can feel present with people as if we were there *[137]*. A unique, creative, and original way for them to express themselves and their music and interact with their fans in an extremely immersive, social setting *[75]*. While I have never done anything about certain sports and interests in the real world, I have experimented with the metaverse by building an important knowledge base *[74]*. Experiencing new experiences painting and sculpture in the video *[114]*. Project a sea onto the bedroom floor and use a real carpet as a virtual flying carpet; or create interactive cute toys that can be a great comfort for sick children *[90]*. Someday in the future, while connected to the metaverse, giving high five to our digitally recreated long-dead ancestors, we will astral projection between virtual fireworks displays, but for now we absorb the fun through motion pictures displayed on flat screens *[138]*. During the pandemic, we used the metaverse regularly to discuss philosophy and met up with the "joyful philosophers’ group." Although the technology was still somewhat cumbersome, we "had a sense of living in a shared world" *[29]*.* What's more, experts think the toys will be much cooler, thanks to the increasingly blurred lines between physical and digital, creating a theme park metaverse where the physical and digital worlds converge [42].

The metaverse is a surrender of the concept of observable truth online that is as real as anything that exists outside of the digital universe [32, 139]. It is understood that the virtual worlds we interact with can be just as real as our ordinary physical world. For example, a conversation in VR is like having a real conversation. Given all the ways virtual worlds can outperform the non-virtual world, people are more likely to be living in a simulation than in the original version of our world [21]. The metaverse, fosters the concept of “reality + ” and broadens our perception of reality [29].

The only limitation of the metaverse is how far people can expand their imagination [76]. By building a fantasy world [140], transporting someone to a fantasy world [141], assuming a different body in VR, visiting Mars, grappling with assassins, and soaring through the sky like a bird [29]. The power to manipulate, change, and regulate one’s physical environment is appealing [35]. Instead of getting into the car with one’s family, you can simply tap a button on your VR glasses and you are instantly taken to a customized digital environment [139]. 'Sitting' together on a bench looking at the moon, 'running' and chasing butterflies one after another, going 'fishing' together behind the house feels liberating [71] or imagining beyond [34] haptic-driven virtual social space for sex [126]. In short, the material world loses its appeal with limitless, believable experiences in virtual reality offered by the metaverse [60].

#### Security policies

Facebooks rebranding to Meta has signaled that it will be more consistent, considerate, transparent, and accountable in managing content [99]. However, it is acknowledge that Meta and the metaverse is still years away from being a full-fledged concept [80]. Meta management seeks to create a culture of proactive engagement of companies against allegations of sexual misconduct and workplace discrimination [40]. As new rules are written for the internet all over the world, the view of being proactive in regulation [66] is developing. Introducing personal limits in some VR applications [32] is an example of regulation. Platforms can also intervene in advance with a culture of proactive participation [31]. Concerns raised by parents, experts and regulators are being considered [39]. Practical measures should be taken, such as the requirement to be over 13 years old to use the metaverse and associated equipment, such as a headset [121], age-appropriate experiences should be developed [39], along with end-to-end encryption of users' data [80].

### Negative content

The metaverse that emerged with Facebook's rebrand and the backlash which resulted in a PR crisis, including the loss of young users [142], a decrease in the number of users [112], cease in being the center of attraction for young people [103] and the disclosures of employees who leaked Facebook information [65, 143]. For this reason, Facebook added the metaverse to its social media ecosystem in order to offer unique experiences to its dwindling young users who are the source of advertising income [38] and to continue its dominant role in SM platforms marketplace [99]. With this emerging new formation, besides adopting the concept of metaverse where people interact in a virtual and physical world, it also triggered warnings that VR will reveal a new wave of problems related to digital platforms [144]. In the metaverse discourse of the experts and regulators who spoke and wrote to theguardian.com, reports of negative opinions arise due to bad experiences from Facebook. The focus and emphasis are striking in some negative opinions. For example, “fearing those who designed this universe, not the meta-universe” [141, 142]. In other words, thinking about the metaverse is the future, but it is a new venue for age-old problems that no one wants to solve [146]. The caveats are to heed when constructing the metaverse, [147] and not allow our perception of reality to be governed [26].

Facebook's dominant market share has also made it difficult to measure and control with its addition to its dominant leadership in SM platforms in the metaverse [39]. It is said that the totalitarianizing of big technologies must be combated [148] in order to prevent large corporations from gradually becoming profoundly dystopian [70]. The lack of transparency [103] of the company that has cost the world like a commodity may be the problem. Given the size of the metaverse questions are raised about how it can be managed and controlled [73]. As in the example of Facebook, the financial losses of millions of users could not be covered in a global outage [149]. It begs the question where people will go if the metaverse is disabled [150]. The mass outage on Facebook platforms, affecting billions of users worldwide, illustrates the dangers of relying only on a few big tech players [41].

Over the past decade, Facebook has sought to capture markets without fully understanding the societies and political environments in which it operates, while not using local experts [99]. It is unclear as to what extent Zuckerberg's view of "learning and progressing while making mistakes" [67] will be impacted by a platform with millions of users as he builds the Meta. It is thought that tech oligarchs like Zuckerberg, with Sauron-like ambitions to own the One Ring to rule them all, seem like the worst choice to take on the responsibility of building a new world [28]. We need to understand why such a large industry has so few competitors [25].

Because technology companies do not have a single regulatory/controller that controls their content for legal and social goodness [119], one way to establish control over the metaverse is to increase control over it, just as with Apple's privacy changes [151]. Dividing Facebook into separate geographic entities will allow new Facebook companies to focus on managing smaller networks [104]. Therefore, it must cooperate with local experts in which it operates by dividing it into small networks within the metaverse. Because no one has yet figured out a way to effectively moderate any place online to keep it safe from abuse, toxicity, and manipulation by bad actors [32]. Similar regulations and independent audit institutions should be established in other sectors [26]. Tech firms should prevent fraudulent advertisements from appearing online [122]. As a result, the need for “fair and open platforms” to prevent creators from holding on too much of their hard-earned money is growing [94], even making it imperative.

#### Misinformation

Misinformation 'miscommunication', aimed at causing non-trivial emotional, psychological, or physical harm, like Facebook's 'threat of serious harm', can be one of the major problems in the [152] metaverse. Much as occurs on other platforms, Facebook has not been able to prevent the spread of negative content about situations that have a significant impact on society, such as vaccination. Algorithms could not filter the content at the desired level [111]. Disinformation about anti-vaccine spread during the coronavirus pandemic period, went viral and users could not be protected from this content [17, 49]. While accusing Facebook of being biased towards conservatism, liberals targeted the platform for its monopolistic tendencies and failure to curb misinformation [130]. It also followed a series of crises in the company accused of spreading fake news around the world, provoking hostilities, and invading privacy [153]. But the company's sometimes indifference has led to questioning of trust, with years of legal litigation [104]that would help reduce misinformation, hate speech, or harm to health on these platforms while simultaneously compelling them. Moreover, it was claimed that the metaverse project was a distraction to get out of the company's PR crisis, risking making the same mistakes as in the company's past [109]. There is a prevailing opinion that investing in Facebook's new concept, the metaverse, without solving the problems in terms of user security and misinformation, is an irrational decision, and the new problems it will create can increase the damage even more. In addition, it is not known how many people were adversely affected by the measures taken against the objections to misinformation or practices that affect people [111].

#### Harmful content

Limiting the spread of illegal content such as terrorist material, images of child sexual abuse and hate crimes, as well as a lack of clarity in definitions of what legal but harmful content is encouraged [150]. Experts warn that children must be protected from harmful content and, for the largest platforms, to protect adults from legal but harmful content likely to include racial abuse and content linked to eating disorders [122]. Omissions such as online abuse, viral gossip, and doxing [34] fail to prevent Meta's failure to keep some users safe and the spread of misinformation [154]. Some measures taken by the company are planning to force the separation of digital avatars from each other after complaints that their metaverse plans could lead to a new wave of online harassment [100]. Although it is reported that 94% of the algorithms have been removed from hate speech [155], the omissions continue. It is compelled to struggle [110]- willingly or unwillingly—to limit the detrimental effect of omissions on its users. It is expected to prevent porn games and applications by combating toxic people and content [156].

#### Algorithms and software

Malicious algorithms continue to be used even though platforms know what they are doing wrong [152]. It is known that Facebook products fuel real-world violence and aggravate mental health problems [157]. Countless new generations of children risk losing their well-being and innocence to uncontrolled algorithms [122]. Algorithms being hidden from the public [155] adds concern that surveillance software may have malicious intent [158]. It allows algorithms to redirect or redirect to harmful content [131]. Due to online trolls and unblocking of anonymous user accounts [122], it is revealed that 72% of toxic discourses came from anonymous accounts [131]. In addition, it is not clear how much content can be controlled in languages other than English (non-English language content) [130]. For these reasons, major technology firms are expected to provide "risk assessments", where they will describe in detail how their platforms might harm users (including the operation of algorithms) and the systems they have to prevent such harm [122]. That's why it is necessary to take precautions against dangerous algorithms [145]. The Meta PR team invests millions of dollars in content moderation, [155] but remains indifferent and incapable of preventing content that will protect users from harmful content, despite warnings that virtual worlds are full of abuse [100]. Adding to all these negativities is the lack of legal practices to prevent the destruction that will occur if there is account [120], which is a part of business and social life, has been used for years. It is a problem to unilaterally block or ban users if they do not comply with their own interests. Deactivating users' accounts with a single command is risky for the user. Are there plans against the impact of a software virus that may occur in the metaverse environment such as user accounts being blocked due to pretending to be someone else [37], inability to protect user privacy [38], their accounts being hacked [25, 112]? It makes the question important.

#### Data

Experts warn that Facebook needs even more foresight and government protection to capture and leverage even more data, and to counter the risk of the space being invaded by big technology [105]. Likewise, they are concerned that data collection technology is infiltrating our most private areas by processing information collected from equipment used in the Metaverse without user consent [90]. To avoid omissions in data collection, Meta can be prevented from collecting certain user data with Apple-style controls. Thus, the meta may be forced to change some of its core advertising business models [159].

#### Equipment

Digital environments based on extended reality (XR) technologies such as VR and AR are still in an early stage of development. There are indications that many of the problems posed by these technologies will also exist in the metaverse [99]. It has an impressive AR display, along with big headphones, annoying headphones, and bad graphics [89], but it's "pretty heavy", doesn't look like regular glasses, and has a very short battery life [35]. Despite the problems such as the size of VR equipment and taking up too much space in the living environment [60], consoles are getting more and more expensive [160]. Users are exposed to environmental hazards [28] while wearing a VR headset or looking at a computer screen in the physical world. VR headsets can often cause havoc in the home. Child safety experts also warn that the headset's lack of parental controls—which would allow parents to block content that could be harmful to children—exposes young users to the threat of abuse on the platform [121].

#### Children

Immersive virtual environments present different and more intense risks for children. Meta has not designed the Oculus headset to be in any way compatible with a security-focused approach [121]. Additional measures should be taken continuously for the development of parental supervision tools for persons under the age of 13, and to use them more effectively in their developed controls. Damage must be prevented before it occurs [26]. Facebook's products are seen as harming children, fueling division, and undermining our democracy [161]. Due to the negative effects of platforms on various applications, especially on the mental health of children, it is expected that companies will prefer to prevent this damage rather than grow [104]. Metaverse equipment security barriers are insufficient to prevent children from enjoying services known to promote child abuse, harassment, racism, and pornography. There are also significant concerns about compliance with the children's regulation [121]. Precautions should be taken to prevent the collection of data of users between the ages of 13 and 15 without express consent and to ensure that it is deleted when requested [157]. Existing problems must be solved before they cause massive new problems [155].

#### Women

The metaverse, which emerged because of Facebook's rebranding to distract from its PR issues, has become another place that is deeply hostile to women [146]. Women are the ones who suffer the most on SM platforms for reasons such as sexual harassment rhetoric and unauthorized nude photo sharing [82]. In addition to the racism problem [162] of some games on the metaverse platform, sexual harassment, and other abuses [163] are evaluated negatively. Firms such as Activision Blizzard, for example, have faced increasing crackdown on allegations of sexual misconduct, including sexual harassment, sexual assault, and gender discrimination [160]. According to some comments on Facebook used to deepen divisions, destabilize democracies, and make young girls and women feel bad about their bodies [128]. The expectation that these negatives will be reflected in the metaverse is strong. In the studies on Teenage Girls, it is stated that the Instagram application harms the mental health of one out of every three teenage girls [150]. Since SM is known to be harmful to adolescent mental health [67], as in these platforms, especially young girls are negatively affected by the content, it is not yet known on which group Meta will have a positive/negative effect [55, 156]. In men, it is reported that it is beginning to cause more men to be drawn into the territory of loafing, pornography, and video games [164].

#### People

What do people lose if they don't use the metaverse? [90]; the negative look that begins with the question “The whole point of VR is to trick your brain into thinking your body is experiencing something… there are too many people who ignore or ridicule the idea of virtual harassment because there are no “real” bodies involved” [146]. There is a risk that the changes that people can create in the perception of reality of the virtual world, leaving people indifferent to environmental problems in the physical world [60].

Services, which are thought to pose a significant risk of harm to individuals [150], add a whole new dimension to violations such as sexual harassment with the immersive nature of VR [146]. Concerns remain about feelings of loneliness and social disintegration. The effects on the mental health of adolescents [91] as well as the effects on children, are of concern. When the fear of being excluded from social environments is added to this, it leaves no choice but to take part in exchanges such as the metaverse [117]. In addition, the problems increase when the previous wrong applications, the processing of faceprint information, access and checking whether the user control controls have been deleted [102]. It creates an environment where bad behavior is personalized and not the natural consequence of existing business models that deliberately push users to be their worst. Therefore, surveillance-oriented business models are needed [116, 151]. Intentional misidentifications [26] and inadvertent user redirects should be avoided.

#### Artists

The metaverse, one of all the virtual places where people spend their time and money [127], is full of failed attempts in entering the world of virtual finance [106]. Risky investments [154, 160], with the effect of crypto money advertising [165]; the desire to direct individuals to crypto money. There are negative approaches such as non-fungible tokens (NFTs) [93], as artifacts exhibited in NFTs do not evoke similar feelings to those in the real world [114]. NFTs also protects artists and photographers’ copyrights but cannot protect them from being underpaid due to intense competition. NFT recipients, on the other hand, have nothing but a URL and some bragging rights [82]. In addition, artists who worry about being excluded [37] from the industry also worry about missing out on opportunities.

#### Society

Similar claims accusing Facebook are causing bias for the metaverse. It stems from the risk that Meta manipulates by creating perceptions in other applications, such as uprising, influencing election results, and many other contexts [157]. Even though Meta harmed society by taking part in some violent acts in the USA, as in many parts of the world, the company's top management remained indifferent [125, 137]. Facebook has repeatedly allowed world leaders and politicians to use its platform to deceive the public or harass dissidents [166]. The institutional version of social media has been accused of undermining democracy from within on some grounds [167]. It aroused mockery and suspicion that a company accused of eroding the cornerstone of global democracy would move to a new metaverse without apologizing for the destruction it caused [157]. For these reasons, because of the destructive power of SM platforms [115], the metaverse was banned in Russia as an extremist organization. It is seen as a problem that it is not clear how the metaverse platform will be used as a tool in a social uprising that may arise anywhere in the world [168]. Even though there are various legal practices regarding these measures in the USA and various European countries, there is no measure related to the positive or negative effects of the metaverse on social mobility, apart from these countries.

It is stated that a gradual trend towards virtual living may eventually lead to new health problems [60]. Choi Jong-ryul, a professor of sociology at Keimyung University, says that while developing an untouchable society has its advantages, it threatens social solidarity and can eventually isolate individuals: “If more people lose their 'sense of touch' due to lack of face-to-face interaction, society will face a fundamental crisis” [91]. Structures like the metaverse are often built by people whose real-world problems are mostly invisible [32]. As in the example of game programmers, the reflection of their personal egos on the content can torture avatars with a personality similar to the friend they get angry with in real business life [126]. Although the use of Metaverse directly affects the lives of many individuals in the society, it can cover the problems reported due to the platform's desire to measure this effect itself [157]. It is stated that Meta has no desire to govern the public in a way that protects them from the consequences of harmful content [168]. It is not preferable to have the necessary tools to mitigate the threats to public health, but not to use them [130]. The constant overflow of new things in the Metaverse [86] makes it a new medium without cultural baggage [30]. What effect the virtual encounters with avatars will have on the nature of the relationships, rather than face-to-face relationships [105], goes beyond guesswork. It reinforces the perception [167] that the metaverse may not have a good future. Another problem is that users of AR and VR devices often stand unassisted, rather than sitting in ergonomic environments. This raises new kinds of health problems, as Jay Kim of Oregon State University explains: "When you stretch your arms away from your body, this creates shoulder strain called gorilla arm syndrome" [169].

### Monkeylearn analysis with sentiment results

In this section, the theguardian.com metaverse contents are listed together with the compound score obtained by VADER analysis and the Monkeylearn classification results and shown in tables. The compound score, which is used as a value in the ranking, is calculated with the VADER model according to the score value of each word in the text in the NLTK dictionary. The resulting score is an average of both positive and negative word scores. Thus, a one-dimensional measure of sensitivity is given to the analyzed text [170]. When we analyze this score together with the Monkeylearn classification results, “Marketing/Advertising/PR” (n = 405) has the most content in the Role Industry classification (Tab.2) and has positive sentiment. “Security/Law Enforcement” (n = 63) and Legal (n = 21) were detected as role negative. Secondly, "Software Development/IT" took place predominantly and its positive perception is quite high. With little content, "Human Resources" shows that other roles have little more to do with the world of the metaverse. The content supports Facebook's metaverse presentation format. There is also a negative perception because of concern about security and legality.

**Table 2:** Metaverse role industry classifier

Row labels	Count	Avg. of compound
Marketing/advertising/PR	405	0.122344198
Software development/IT	220	0.333531818
Art/design/entertainment	97	0.369595876
Security/law enforcement	63	− 0.124130159
Non-profit/volunteering	44	0.221759091
Banking/loan/insurance	25	0.207444
Legal	21	− 0.296161905
Medical/healthcare	18	− 0.0501
Sales/customer care	17	0.044152941
Installation/maintenance/repair	15	0.11866
Writing/editing/publishing	14	0.100871429
Beauty/wellness	11	0.300718182
Sports/fitness	10	0.31725
Science/research	10	0.13548
Hospitality	9	0.3166
Management	8	0.152325
Restaurant/food services	6	0.1155
Accounting/finance	6	0.25265
Architecture/drafting	4	0.314425
Education	4	− 0.28045
Travel/transportation	3	0.144433333
Business development/consulting	3	0.500766667
Manufacturing/production/logistics	3	0.303533333
Real estate	2	0.51865
Human resources	2	0.3793
Product/project management	1	0.1779
Skilled trade	1	− 0.5574
Facilities/general labor	1	0.3612
Grand total	1023	0.177511046

In the event classification of the texts (Table 3), “Science & Technology” (n = 407) and “Entertainment” (n = 285) have a positive perception by creating the most content. The title "Health & Medicine" has a negative score. It can be said that these results are seen as technology and entertainment in the early stage of the metaverse, and it has not yet gained a place in other event classes.

**Table 3:** Metaverse events classifier

ow labels	Count	Avg. of compound
Science & technology	407	0.172897297
Entertainment	285	0.229042456
Arts & culture	191	0.15664555
Movements & ideologies	36	0.048980556
Family & children	31	0.109503226
Businesses & industry	25	0.264636
Health & medicine	24	− 0.234025
Beauty & fashion	14	0.656007143
Food & drink	10	0.06869
Grand total	1023	0.177511046

In the Metaverse Monkeylearn Business Classifier (Table 4) results, “Recreational” (n = 554) was the most and positive sentiment, while “Legal” (n = 1) was the least content. “Government” (n = 111) negative sentiment score has. The findings are perceived as platform entertainment in the metaverse business classification. It is an important result that negative content and discourses about the government are negative. It can be said that there are concerns arising from the effects on metaverse individuals and their social structure.

**Table 4:** Metaverse business classifier

Row labels	Count	Avg. of compound
Recreational	554	0.221808845
Service	149	0.033453691
Government	111	− 0.02944955
High tech	64	0.3184625
Media	42	0.212535714
Arts	32	0.299934375
Consumer Goods	30	0.26989
Finance	19	0.244415789
Agriculture	5	0.42832
Non-profit	4	0.3639
Medical	4	− 0.3461
Educational	3	0.3607
Corporate	2	0.33725
Manufacturing	2	0.3399
Transportation	1	0.6486
Legal	1	0.0516
Grand total	1023	0.177511046

## Conclusion

The Leximancer analysis shows “facebook, games, and platforms” as the main theme in theguardian.com metaverse content. For this reason, it was concluded that games play an important role in the development of the metaverse, and platforms, specifically, social media platforms which foster networking and sharing, will have an impact on the development and future of the metaverse. In the results of sentiment analysis, negative perceptions are the problems caused by Facebook and other SM platforms, as well as the negative statements that arise because of the ex-employee leaking company information. Time is required for Facebook to institutionalize and create its own culture. Transparency is needed as to what policies the company will pursue against the societal impacts of a large project like the metaverse. The effect of the algorithms on users is not fully known, and there is a lack of clarity. Although the company has announced that it will take measures in cooperation with the academic world, it is a mystery how to monitor the billions of codes created by thousands of software developers. Presently, there are nothing more than statements about how data is processed and used. It can be said that countries other than the US and UK do not even have an idea about how to legally control a platform that is estimated to have billions of users in the future.

Monkeylearn classification findings show that the general trend of discourses is perceived as just games and entertainment in the metaverse, however, the goals of the metaverse go far beyond that. The metaverse in transforming the world as we know it and there are no longer any boundaries between the physical and virtual world which are now seamless integrated. The challenge that the metaverse faces is to establish a new universe that will cover not only entertainment but also other life-related areas, including, ecommerce, work, hobbies, and study. Therefore, it will take time for the targeted objectives to be realized and to cover other sectors. In addition, there is a concern that Facebook and other SM platforms will encounter any problems that harm users or generate new problems in the metaverse. This causes biased approaches in the newly created metaverse. Scientific studies on SM platforms are used as evidence to create this bias. It can be said that negative experiences in the past give clues about what will happen in the future. When deducing from the negative sentiment results in general, it can be concluded that the Facebook brand name change to ‘Meta’ shadows a huge investment of $10 billion (insert reference here) into the successful formation of the metaverse. This amount is 10 times that paid to purchase Instagram (Insert Reference here).

In addition to the possibility of metaverse failure, the risk of new technological products coming onto the market due to increased competition may cause investors to implement different strategies at any time. There has not been a discourse that informs the public about what the social and individual effects of these strategies might be. Billions of people and countries have been involved in this large-scale project but, arguably, have little idea about what the positive/negative effects of this project will be. If it weren't for theguardian.com metaverse negative rhetoric, as indicated in this present study, along with Facebook employees’ disclosure, controllers, and a few experts' rhetoric, we might not even be aware of the harmful effects experienced by the society of this fast-evolving virtual world.

Furthermore, this study had found negative attitude towards technology oligarchs evident in theguardian.com content. In particular, the content of the metaverse constructors is not related to real world problems, and their own prejudices and consciousness are reflected in the positive/negative sentiment about the metaverse. It can be said that there is nothing more than inferences about the problems that may arise in the real world during the development of the metaverse. There is such a thing as experiencing by living. Late detection of the negative effects of metaverse applications on users, as in other SM platforms, may increase the destruction of the effect on large masses. For this reason, both the company and the authorized government institutions should take precautions before problems arise, by implementing proactive policies against such negativities. As another precaution, the idea of metaverse users using local experts to control the content produced in hundreds of languages other than English, is being voiced. Creating a controllable structure by dividing the company into smaller networks in separate regions stands out as one of the solutions. For example, with some programmatic restrictions, Meta is allowed to collect certain user data. This forces the company to change some of its business models. It is made indirectly controllable from outside on the metaverse. Based on this application, control software on metaverse equipment can protect those most vulnerable, including children and women, from harmful content, harassment, overall negativity, and bad speech.

As indicated by positive discourse in this study, it is noted that with the emergence of the metaverse, big companies, especially technology companies, entered the investment race. In addition to acquisitions costing more than billions of dollars (e.g., Microsoft is to pay almost $70bn to buy Activision Blizzard), countries have begun to enter the metaverse environment. For example, in Europe, new projects such as "euro metaverse", have already started. Many metaverse-related new jobs and professions have begun to emerge. Job opportunities were created for 10,000 new employees in Europe. New professions and business models began to emerge. The metaverse, which created a perfect positive storm even in the pandemic, has helped companies in many industries to thrive despite the restrictions. In addition to exhibiting their own products on the platform, they had the opportunity to promote and market many new products. People who could not leave their homes due to restrictions spent their time in games, entertainment, and chat rooms, and they socialized virtually by meeting new people online. These results enabled technology companies to make significant profits. Companies and users multiplied their earnings by producing virtual assets. Even with NFTs, their creations were able to protect unique beings. New ways of making money have opened for all metaverse stakeholders. It has enabled companies to change their business models and consumer strategies. For a company like Facebook, it has meant moving its activities to the virtual environment, and environmental damage began to decrease. Even the way employees do business began to change.

Moreover, the metaverse paved the way for the development of new technologies such as 3D internet, web 3.0. It enabled the renewal of VR/AR equipment and increased their market share. The developments in equipment have started to optimize technological opportunities in a way that will increase the quality of life and earnings for people, users of the metaverse. The metaverse has started to become the new entertainment center for young people and children due to the colorful, lively, and entertaining environment in its content. The virtual world now offers its users opportunities to discover new experiences and increases their creativity. They extend their physical life with avatars. The metaverse offers new emotions that people had not felt before in the physical world. The foundations of digital society have begun to strengthen by enabling the meeting between new people and the formation of groups with the opportunity to move far away with their bodies with avatars. Regardless of people's ethnicity, language, and religion, new cultures and concepts are formed. As a result, the metaverse becomes one of the important players in the new digital public space. New concepts such as digital society and public space are getting stronger.

The metaverse equipment allows exploration of body language and access to new types of data. These data make it easier to determine people's behavioral norms. It can change the information of companies in customer databases by expanding the quantity and quality of data. The metaverse also uses its own AI models for these processes and can do more with less data by constantly training its algorithms. The metaverse, which also paves the way for the perception of reality to be redefined and experienced, makes many new actions that cannot be done in the physical world possible. While doing this, investments are made to ensure the safety of users on the platform, and a proactive company culture is developed. It uses new business models powered by AI capabilities to continuously combat crimes against humanity, such as human rights, sexual abuse, and racism.

In addition, it can be said that the method used in the study makes a practical contribution in terms of drawing meaningful results from unstructured large textual data with computational qualitative analysis with NLP facilities. It can be said that consistent findings have been obtained by processing, visualizing, and measuring metaverse development from selected online textual data beyond traditional qualitative analysis methods. Considering the size of the processed data, the contribution of AI facilities to scientific studies is important. This study is also a good example of analyzing text content using the Orange 3, Leximancer, NLTK, Monkeylearn AI software tools together. Specific contribution is anticipated to future studies in social sciences which will be carried forward with NLP using AI.

The study is limited in that it cannot reveal the metaverse discourse in different geographies, as it analyzes the content of the western-sourced mainstream media organ. In future studies, the analysis, findings, and the conceptual relationships in the Leximancer concept map can be the subject of research. Considering the positive/negative aspects of the emerging metaverse, authorities can make various applications. Finally, the metaverse is the world based on the post-modern movement, supported by decentralized technology, created by lonely people, and individualizing people. We can define it as the first virtual system where individuals do not want to be controlled, where the choices of individuals, not the majority, are decisive, different feelings and experiences are experienced, and both positive and negative aspects are present.

## Data Availability

Not applicable.
